# Translational Research for Occupational Therapy: Using SPRE in Hippotherapy for Children with Developmental Disabilities

**DOI:** 10.1155/2017/2305402

**Published:** 2017-02-19

**Authors:** Deborah Weissman-Miller, Rosalie J. Miller, Mary P. Shotwell

**Affiliations:** School of Occupational Therapy, Brenau University, 500 Washington Street SE, Gainesville, GA 30501, USA

## Abstract

Translational research is redefined in this paper using a combination of methods in statistics and data science to enhance the understanding of outcomes and practice in occupational therapy. These new methods are applied, using larger data and smaller single-subject data, to a study in hippotherapy for children with developmental disabilities (DD). The Centers for Disease Control and Prevention estimates DD affects nearly 10 million children, aged 2–19, where diagnoses may be comorbid. Hippotherapy is defined here as a treatment strategy in occupational therapy using equine movement to achieve functional outcomes. Semiparametric ratio estimator (SPRE), a single-subject statistical and small data science model, is used to derive a “change point” indicating where the participant adapts to treatment, from which predictions are made. Data analyzed here is from an institutional review board approved pilot study using the Hippotherapy Evaluation and Assessment Tool measure, where outcomes are given separately for each of four measured domains and the total scores of each participant. Analysis with SPRE, using statistical methods to predict a “change point” and data science graphical interpretations of data, shows the translational comparisons between results from larger mean values and the very different results from smaller values for each HEAT domain in terms of relationships and statistical probabilities.

## 1. Introduction

Translational research is often defined as translating research done in the laboratory so it can be applied in clinical practice in medicine. Translational research also applies to the adoption of best practices in the community. For the entire history of occupational therapy (OT), there have not been sufficient statistics to “translate” OT and its perspective into a relevant scientific viewpoint [1]. Traditional statistical results from large datasets have not been able to show that engaging in meaningful occupation has statistically significantly improved a client's situation. However, translational research in occupational therapy may now be defined by new statistical and data science methodology available to analyze the data. Until recently, occupational therapy outcomes have been analyzed by means of larger data, such as *t*-tests and randomized controlled trials. These types of analyses are uniquely suited to public health and medicine but are not appropriate for the usual approach of occupational therapy treating one client, or a small group, at a time to determine the occupation meaningful to the client and design therapy accordingly. A goal in occupational therapy in this paper is to combine resources from treatment data with a new single-subject and small group design in statistics. This new single-subject design may also be used as small datasets in data science to promote enhancements in graphical analysis of prevention and therapies. In occupational therapy, translational research aims to “translate” findings in statistical and data science research results from larger group data into meaningful comparative results from smaller single-subject datasets that have been extracted from a portion of the group data and that influence clinical observation as part of occupational therapy practice. Translational research can implement “data to practice” outcomes to produce new treatment options for patients.

A fundamental model for this approach is the use of the new semiparametric ratio estimator (SPRE) as a single-subject statistical and small data science model to define, analyze, graph, and predict occupational therapy data to provide a pathway to meaningful treatment. SPRE, as the semiparametric ratio estimator discussed by Weissman-Miller et al. [2], in Weissman-Miller [3], and in Weissman-Miller [4], provides a statistical science model to determine the “change point” where the participant adapts to treatment. In the SPRE model, the change point is derived from a backwards stepwise ordinary least squares regression, which provides minimum bias. The change point is determined from the highest or lowest *F* statistic, according to Weissman-Miller et al. [2] as

(1) $$\begin{array}{c} F=t^{2}=\frac{MSReg}{{\hat{\sigma }}^{2}}, \end{array}$$

where *F* is the *F*-distribution, also known as Snedecor's *F*, which is a continuous probability distribution used here to determine the highest or lowest *f* statistic from the ordinary least squares regression, *t*^2^ is Student's *t*-distribution that is a continuous probability distribution that arises when estimating the mean of a normally distributed population in situations where there is a small sample size, MSReg is the mean square due to regression, and ${\hat{\sigma }}^{2}$ is estimated population variance.

The result is associated with the relevant distributional *p* value [3] at the value of the time interval in the session number. From the change point, nonlinear estimates are given in the SPRE model using a new response function given from the cumulative distribution function of the Weibull distribution. These point estimates are given using a ratio of the Weibull distribution times the prior estimated outcome [3]. The new response function *G*_*k*_, *τ*(*t*) is given as

(2) $$\begin{array}{c} \left(\;1-e^{{-\left(t/\tau \right)}^{k}}\;\right), \end{array}$$

when *ε* is the number 2.71828, the base of the natural logs, *t* is time, *τ* is the value of the change point, in this SPRE analysis (using the hereditary integral), and *k* determines the curvature of the distributional predictions.

Then the point estimation may be given as a ratio (*R*) of the Weibull distributions times the prior outcome.

The predictive equation from Weissman-Miller et al. [2] is then

(3) $$\begin{array}{c} {\hat{\theta }}_{t}=R\cdot \theta_{t_{i}}, \end{array}$$

where ${\hat{\theta }}_{t}$ is outcomes beyond *τ* that are point estimates, *R* is ratio of Weibull distributions (varying by *t* = time), and *θ*_*t*_*i*__ is population parameter at the prior time.

**Figure 1:**
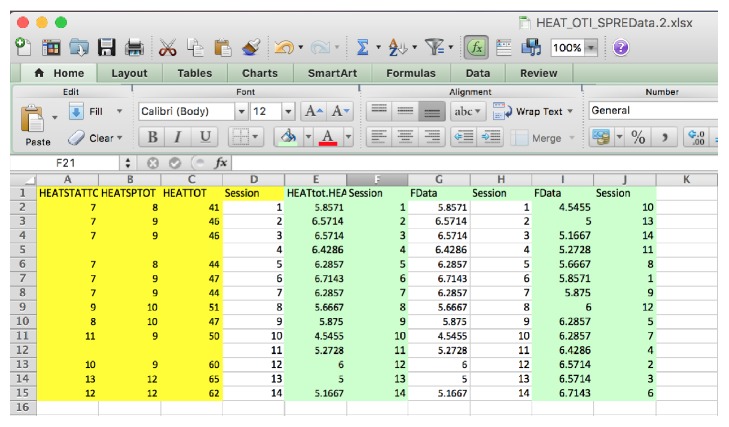
Excel (CSV) data for static posture of Partic.c for SPRE from raw data to arranged data.

Any OT data will fit the SPRE model if the sessions, a minimum of 13 to 14, are reasonably evenly spaced. The OT data must be taken from a measure that will yield results for the specified research question. Then, the raw data is entered into Excel and finally saved as a comma separated file (CSV). This type of file can be read directly by the SPRE program written in the R software language. The initial worksheet data is saved as an Excel worksheet. The final data for each participant is saved in a new worksheet as a CSV file. It can be seen in Figure 1 that the data headings for the SPRE R code are labeled FData and Session for the participant Partic.c, taken from an Excel file of this participant's data. These results will vary according to the input data, but the format for the SPRE R program is always the same, where the “FData” and “Session” are highlighted together and arranged in ascending or descending order following the order of the original raw data coded in Excel. Then, the session numbers are always arranged together with the original raw data that allows for accurate predictions of the change point [3].

*Context for This Study.* This study took place in the context of exploring the effectiveness of hippotherapy practice and a newly developed measure called the Hippotherapy Evaluation and Assessment Tool (HEAT). Hippotherapy is “therapy with the help of a horse” derived from the Greek word hippos, meaning “horse” [5]. Classic hippotherapy treatments, used in the rehabilitation of musculoskeletal, neuromuscular, and cardiopulmonary dysfunctions, use the movement of the horse and promote the patient's response to its dynamic movement [6]. The purported benefits of hippotherapy impact multiple body systems [7]. Literature identifies common tools used to measure multiple body system deficits ranging from static posture to dynamic motor behavior, sensory processing, and psychosocial/behavioral difficulties. Postural effects of hippotherapy are frequently evaluated using the* Pediatric Balance Scale *(PBS) [8] or the* Sitting Assessment Scale* (SAS) [9]. The* Gross Motor Function Measure* (MFM) [10] is often used to measure dynamic motor behavior. The* Sensory Profile* (SP) [11] and the* Sensory Processing Measure *(SPM) [12] assess sensory processing patterns. Psychosocial and behavioral domains are frequently evaluated with the* Pediatric Evaluation of Disability Inventory* (PEDI) [13]. The assessment used in the research described in this paper is the HEAT (*Hippotherapy Evaluation and Assessment Tool*) as given by Malone et al. [14].

*Data Organization in SPRE for Statistical and Data Science.* It should be noted that while any OT data can be input into Excel, the data is often statistically transformed depending upon the OT practice area for analysis in SPRE. For data in the HEAT measure [14], the raw data in Figure 1 was analyzed as

(4) $$\begin{array}{c} \text{HEATTOTAL}/\text{HEATStaticPosture}. \end{array}$$

The data in Figure 1 are data for Partic.c, computing the ratio using (4) and arranging the data. These results will vary according to the input data, but the format for SPRE R program is always the same.

The important part of using SPRE as statistics and data science is that each participant is their own control, which means that the efficacy of data no longer depends on having a homogeneous participant pool. In this case, participants with varying comorbidities can be analyzed separately as single-subjects. Of course, total data can be taken and then analyzed for a small group. This approach, combining single-subject data, is much more effective for occupational therapy than pre-post analyses involving participants with comorbidities and very useful for translational research.

The usefulness of SPRE can also be seen in this paper where the participants have been diagnosed with comorbidities, because these participants can all be included in the same study, while they are analyzed separately. To facilitate the use of SPRE in a clinical setting, an “R” statistical program [15] has been written to compute data results collected from participants and prepared using Microsoft Excel [16]. This “R” program includes predictions and comparative graphical analytical tools that are a mainstay of data science. The question arises: what is the difference, in this context, between statistical and data science? An answer is that statistical science is fundamentally about a model. Data is trimmed and outliers are often discarded altogether to better fit the model. An example of this is the linear regression model, where missing data is not considered in the dataset and data outliers may be discarded as demonstrated in Weisberg [17]. In the small datasets used in occupational therapy, and particularly in single-subject design, there is a limit to the number of sessions that can be provided within time and duration of treatment constraints. Discarding sessions with missing data or with data that seems “outside the norm” provides too little data for analysis, according to Weissman-Miller and Holmes [18]. Furthermore, the whole of the data represents the participant's actual results. Discarding any of that data to fit a model may be considered in the same way as discarding some of the occupational therapists' descriptive notes on a participant because they do not fit the therapists' own assumptions. Using SPRE, missing data may be imputed using an approach described by Weissman-Miller and Holmes [18], where an error function for the data is derived. In doing this, the translational data and results are taken from the participant's actual complete data.

## 2. Materials and Methods

### 2.1. Design of the Study

This SPRE analysis is part of a research project which contributed to validity and reliability of a newly developed Hippotherapy Evaluation and Assessment Tool (HEAT). The HEAT is intended to provide a common tool for therapists to measure ongoing progress as well as outcomes in hippotherapy practice. In the original IRB approved protocol from which this analysis is taken, a nonexperimental repeated measures study used data collected from 21 children receiving hippotherapy services in order to establish predictive validity of this new hippotherapy measure (HEAT). Research questions included the following: (1) Do scores on the HEAT show a statistically significant change over repeated measures? (2) Based on the *F*-statistic, can researchers predict the number of treatment sessions a client will need before they reach a “plateau” in progress? Once data was collected, it was analyzed using several techniques for all the data, such as a Kruskal-Wallis ANOVA (nonparametric ANOVA equivalent), the SPRE model using an ordinary least squares (OLS) linear regression, and a stepwise prediction method based on a ratio of a Weibull parametric cumulative distribution as given by Weissman-Miller et al. [2] and Weissman-Miller [3].

### 2.2. Summary of the Original Hippotherapy Evaluation and Assessment Tool (HEAT) Measure

The Hippotherapy Evaluation and Assessment Tool (HEAT) was developed by the third author of this paper (Shotwell) in response to the need for a comprehensive assessment to measure the outcomes of hippotherapy and used as part of graduate thesis research [19]. Using the literature in hippotherapy as a guide for what items should be on the HEAT, Shotwell, along with her graduate students [19], found that 45% of the literature discussed dynamic motor performance as an outcome of hippotherapy, 20% of the literature showed improvements in static posture, 20% of the articles represented changes in sensory processing, and the smallest percentage of outcomes (15%) was represented by psychosocial or behavioral changes. The final version of the HEAT is a 100-point measure containing four domains: (a) dynamic motor behavior; (b) static posture; (c) sensory processing; and (d) psychosocial/behavioral performance.

The overall purpose of the recent Malone et al. [14] research study was to further explore two forms of criterion-related validity of the HEAT in terms of (a) predictive validity within a repeated measures design and (b) further analysis of HEAT's concurrent validity in comparison to other “gold standard” measures. The repeated measures design portion provided an opportunity to apply the SPRE model to small group research.

### 2.3. Sampling and Recruitment

Malone et al. [14] used purposive sampling to recruit 21 children, male (*n* = 8) and female (*n* = 13), between the ages of 2 and 19 years who were engaged in hippotherapy services. According to Portney and Watkins [20], purposive sampling is a technique researchers use to select subjects on the basis of specific criteria. In this study, researchers wanted to test a variety of subjects with specifically different degrees of limitation. Purposive sampling was well suited to analysis using SPRE for a heterogeneous single-subject design. Children who had participated in hippotherapy were recruited from outpatient rehabilitation facilities and barns in the Southeastern US, to ensure familiarity with horses. Inclusion criteria required that participants speak English, unless a translator was available. No targeted gender, ethnicity, or diagnosis group was excluded. Attempts were made to recruit children with diagnoses typically seen in hippotherapy practice including autism; cerebral palsy; attention deficit hyperactivity disorder; Down syndrome; and other genetic disorders.

### 2.4. Data Collection and Analysis of the HEAT Sample Data

Data were collected over the course of 14 weeks during hippotherapy sessions using the HEAT. The Pediatric Evaluation of Disability Inventory (PEDI) was also administered during the first and last sessions in order to explore concurrent and predictive validity. Paired *t*-tests were performed to investigate HEAT's ability to show change over time. This quasi-experimental repeated measures study used data collected from 21 children receiving hippotherapy services. The quasi-experimental design was employed for this study because there was no random assignment or comparison group. To establish predictive validity of the HEAT, four research questions provided the framework for data collection from each participant. These questions included the following.

(1) Is there a difference in HEAT scores pre-post? (1a) Do scores on HEAT show statistically significant change over time? (1b) Does hippotherapy intervention show changes in functional behaviors as measured by the PEDI scores? (2) Is the HEAT a valid measure with respect to comparison with an “occupation-based measure” such as the PEDI? Once data was collected, statistical analysis consisted of (a) paired *t*-tests, (b) semiparametric ratio estimator (SPRE) model, and (c) exploratory correlations. Since children participating in hippotherapy are often unlike each other, a group design comparing them to each other would be flawed. It would be more appropriate that the client be their own control and their pretest scores compared to their posttest scores. Since the HEAT measure is intended for repeated measures, stability over time is explored as shown in Malone et al. [14].

Pre-post differences show that three of the four domains of the HEAT had statistically significant results except for static posture (*p* = 0.071). The paired *t*-test results in Table 1 give the statistical significance and an overall summary of the results of HEATTOTAL and each subdomain. The majority of the *t*-tests exploring differences in each domain in the HEAT were statistically significant. While this is useful information showing the sufficiency of the HEAT measure, this type of analysis does not provide a look into the “black box” of therapeutic result over time. From the overall result in Table 1, for 12 participants from this at risk dataset who completed sessions 1 and 14 for the pre-post sessions [14], we can determine what happened, but not why the results may have changed over time, or how the therapy may be improved. An advanced statistical technique, the semiparametric ratio estimator (SPRE) was conducted to further determine the HEAT's sensitivity and stability. Individual analyses by the SPRE model provided further comparison and insight between observational notes and numerical data on HEAT resulting in translational research of the data.

### 2.5. Design of the Study for Translational Research

Data obtained through the data collection process using the HEAT measure was entered in SPSS [21] to analyze 12 participants' pre-post results using paired *t*-tests. These results were statistically significant except for HEAT static posture (*p* = 0.071). Other statistical measures were used to analyze data for the total participant sample, *n* = 21. The next question addressed was what kind of change the participants' scores showed over time using the SPRE program written in R from Excel software. The R Development Core Team [15] computer software language provides a wide variety of statistical and graphical techniques that provide a basis for comparing individual results both numerically and graphically in statistics and data science. For the translational analysis in this paper, a subset of the sample (*n* = 5) was initially selected to investigate the potential for determining comparative results. The five participants were selected by the completeness of their datasets for use in SPRE analyses. In addition, the biostatistician was blinded to the identity of each participant, to reduce possible bias. The Excel data for each of these five participants was analyzed using the R program for SPRE. This program is used through R Studio [22]. To read the data, the Excel program must be saved as a “csv” or data file. Then, listing the dataset and sourcing the R program will read the dataset, for example,

  Dataset <- read.csv(“Falls.d.Final2_2014.csv”)
  source(“test175.R”)

The data name in the parentheses is your data. An illustration of this method in R Studio is given in Figure 1.

### 2.6. Analysis of a Subset of Selected Participants

Of the five randomly selected participants, four had had 14 sessions and one had had 13 sessions. Four participants were missing some data. The use of SPRE as a single-subject design model easily allows for the imputation of missing data. In this analysis, data was imputed using an approach given by Weissman-Miller and Holmes [18], so that “complete” datasets were analyzed in SPRE to derive the “change point” for each of the 5 participants and the predictions of future efficacy of the treatment. In this study, the error estimates for missing data varied between 0.0005 and 0.0526 for the participants. The first consideration is to minimize the order of magnitude of the experimental data as much as possible before using LERP (linear interpolation) to fill in the necessary data between 2 existing raw data points. Therefore, the interpolation should be made almost at the last step of data preparation, where the order of magnitude of the ratio from (4) will be small. The most important part of this analysis is given by the error function. The error function for these data points is derived from Parnell [23] and computed from the second derivative of a cubic polynomial function derived from numerical analysis. The details and a complete analysis using data from fall prevention in elders are given in the paper by Weissman-Miller and Holmes [18]. The fundamental question is whether imputation of data, using a small local linearized segment of the original data points, distorts the data fit of the SPRE model. Since many, if not most, single-subject designs for small datasets have missing data, the basic idea in this analysis is to provide an error function, associated with each imputed data point, which can determine any potential for distortion. This assumption provides an answer in each case, not whether or not to impute data, but if the imputed data points have sufficiently small error functions. Then the SPRE model results can be considered sufficient, as in this analysis of the HEAT data for each participant.

The total data for each participant, across all domains, was run through the SPRE software program, producing numerical and graphic results. Then, each of the subdomains in the HEAT measure was analyzed for each participant. The results of these analyses for one of the HEAT domains were surprising and led to the interpretation of translational research in occupational therapy in this paper.

## 3. Results

**Figure 2:**
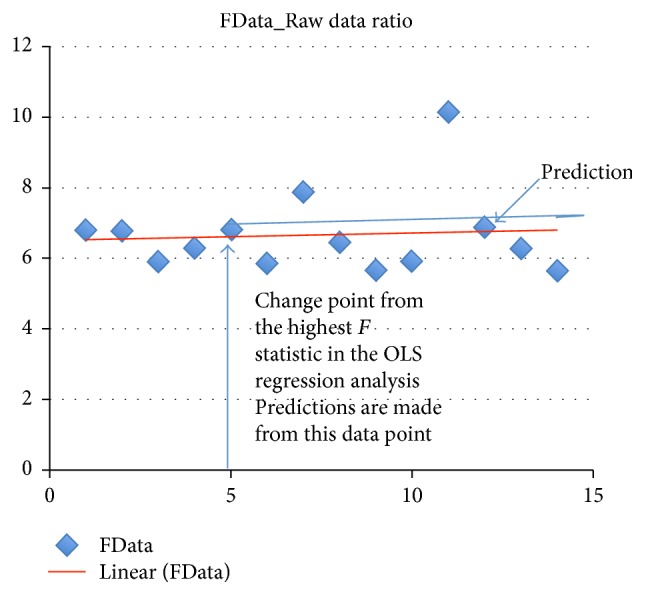
Partic.c_Sensory Processing plot of regression on data with change point and prediction.

A graphic representation of the SPRE model, using a single participant's data from one domain, shows the change point in Figure 2.

This is a regression problem in which the expected value of the dependent variable is assumed to have a different functional form in specific neighborhoods of the explanatory variable space. According to Weissman-Miller [3], in the SPRE model, the determination of the change point is a structural change that shows the dynamic nature of participants with comorbidities. The highest or lowest change point from which this participant's predictions are made is at session 5. The ability to calculate *p* values for the data points from the regression analysis and make predictions is a unique strength of the SPRE model.

### 3.1. Results of the HEATTOTAL Variable

The HEATTOTAL variable is analyzed using SPRE. The outcomes for the total scores are positive for four individuals and have a normal distribution of the data, as shown by the scatter of the residuals, except for one participant. Because there is only one negative single-subject prediction among these five participants, the question is as follows: What is the total number of negative predictions among the 21 final participants' scores? In this study there were only three negative HEATTOTAL scores in the entire dataset. Therefore, the mean data from these three would have a very small or negligible impact on the statistical significance of all the HEAT data analyzed as a whole dataset. In addition, when looking at one of five negative results, that is, still a relatively small impact when considering the mean data for all five participants, the paired *t*-test results give the statistical significance and an overall glance for the results of HEATTOTAL and each subdomain. While this is useful information as to the sufficiency of the HEAT measure, this type of analysis does not provide a look into the “black box” of therapeutic results. From overall results in Table 1 from Malone et al. [14], we can determine what happened, but not why the results may have changed over time, or how the therapy may be improved.

The results of HEATTOTAL for the random five participants are given in Table 2. Individual participants' data were analyzed using the SPRE as described by Weissman-Miller et al. [2] and by Weissman-Miller [3]. The participants have been described as Partic.a, and so on.

The value of being able to analyze the data using SPRE is that if the mean of all the data from all participants is analyzed and it is positive, there would also be a mean change point and *p* value. This type of analysis would validate the HEAT measure as a whole. However, SPRE can also be used on individual totals as well as each domain for each participant as shown in Table 2. The values for each participant are small for each domain, and the *p* values do reflect the statistical significance of the level of function for that individual. However each *p* value would not reflect the significance of the measure for all domains for all participants.

### 3.2. SPRE Analysis of Individual Participants in the Static Variable Domain

Individual participants' data were analyzed using SPRE. A summary of variable outcomes is given in Table 3.

It can be seen that there were two negative predictions among the five participants. Analyzing only the total data from one variable is quite different from taking the total of all of a participant's data. In this case, we can see that the participant named Partic.e is still negative, but the participant named Partic.c is also negative. When all of Partic.c's data was totaled, her prediction direction was positive, as can be seen in Table 2. The predicted difference for all the total data for one participant can be quite different when compared to the total of only one domain of data. In this way, translational research in occupational therapy begins to be implemented by “translating” comparative findings in statistical science research results into more meaningful occupational therapy practice and outcomes for each single participant. In this analysis, the analytical tool SPRE shows that Partic.c's static posture may be a problem that should be reviewed by the therapist.

### 3.3. SPRE Analysis of a Single Participant for All Domains

Partic.c's individual data were analyzed using SPRE. A summary of variable outcomes is given in Table 4.

An analysis was performed of each domain: static posture, dynamic motor behavior, sensory processing, and psychosocial/behavior for the participant (Partic.c). Her static posture prediction was negative, while the predictions for the remaining domains were positive. The comparative results show that the residuals of the data for the sensory processing domain are reasonably normal with outliers and the predictions are positive in this case. The outliers of the sensory processing domain may indicate that sensory processing is a problem for this participant. These relationships should be further investigated both graphically and statistically to determine the necessity for specific and targeted occupational therapy treatment.

### 3.4. SPRE Numerical and Graphic Results for the Partic.c

**Figure 3:**
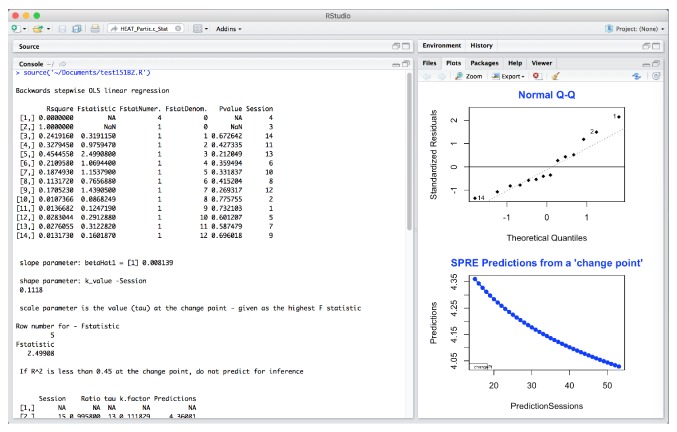
HEAT standardized residuals analysis for Partic.c _Static posture in SPRE.

The SPRE analysis in Figure 3 is for Partic.c, for static posture. The plot in the upper right side of the figure, labeled “SPRE residuals,” shows standardized residuals against theoretical quantiles of the data for this variable.

**Figure 4:**
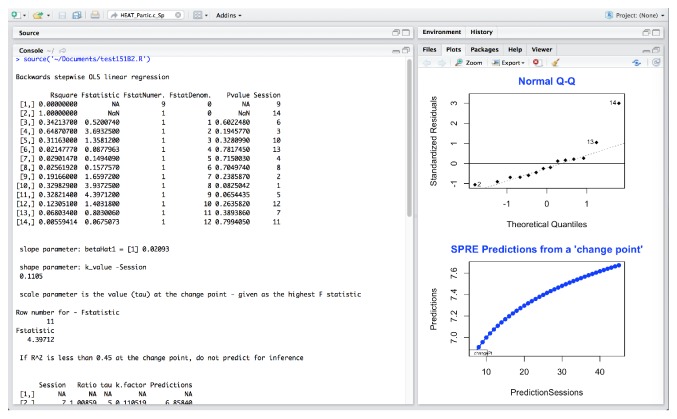
HEAT standardized residuals analysis for Partic.c _ sensory processing in SPRE.

In Figure 4, the sensory processing data is analyzed in SPRE and plotted using the same format. The plots have a very similar shape although the outlying standardized residuals are different.

**Figure 5:**
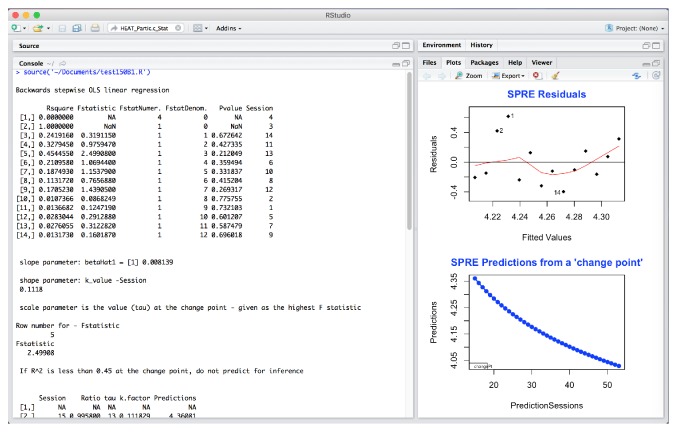
HEAT residuals analysis for Partic.c _ Static posture in SPRE.

In Figure 5, the upper right hand plot plots the residuals against the fitted values for static posture.

**Figure 6:**
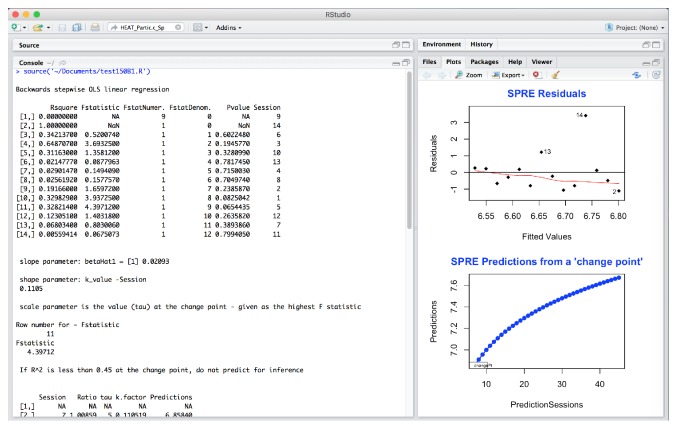
HEAT residuals analysis for Partic.c _ sensory processing in SPRE.

In Figure 6, the upper right hand plot plots the residuals against the fitted values for sensory processing.

These four plots show very different information. In Figures 3 and 4, the normal Q-Q plots have very similar shape. While Figure 3 shows a reasonably normal distribution with 2 potential outliers, Figure 4 also shows much of the data following the normal curve but with more defined outliers. Looking at these two plots from a data science perspective, the comparative resemblance of the shape indicates a possible relationship between the static posture and the sensory processing variables.

However, in investigating the data further, Figure 5 now indicates that these residuals from the regression analysis are from data in a normal distribution, with the same 2 potential outliers. However, the residuals in Figure 6 point to a real problem in the residuals of the data analysis results for this participant in this domain. This type of plot shows that the residuals from the data for this variable are not randomly scattered and therefore not from a normal distribution. Furthermore, the variance is not constant (when these residuals are shaped like a cone). This means that when one plots the individual error against the predicted value, the variance of the error predicted value should be constant. In fact, the SPRE residuals in Figure 6, which show nearly a straight line below 0.0, are so unlike the randomly scattered shape of the residuals in Figure 5 above and below 0.0, that it indicates a problem with the sensory processing for this participant. Additionally, the negative slope to the prediction for static posture in Figure 3 together with a normal distribution of the residuals of the data indicates a potential problem with static posture for this participant. Likewise, the extreme nonnormality of the residuals in sensory processing in Figure 6 indicates that sensory processing for Partic.c is a problem, even though the predictions are positive.

The findings from Figures 3–6 taken together indicate a problem in each of these two domains for this participant. This indicates a potential problematic therapeutic relationship between static posture and sensory processing in this case, based on comparative findings and anomalies from a statistical and data science approach to identifying a therapeutic problem for this participant.

## 4. Discussion

In this paper, translational research findings are applied from a combination of numerical methods in statistics and graphical methods in data science. This is particularly true when the use of the SPRE model has proceeded from the analysis of the total sum of the HEAT measure to the analysis of the individual variables for each participant. The result of this translational process from analyzing the larger mean to the specific and personal data has been to uncover a relation between two of the variables in that participant's responses and to determine potential therapeutic needs.

Following these discoveries in Partic.c's outcomes, a connection was considered between static posture and sensory processing, as both constructs take into account proprioception and vestibular function. According to Case-Smith [24], developing the vestibular-proprioceptive-visual connections provides the beginnings of postural control and continues to refine, resulting in further development of balance and fluidity in dynamic postural control. This study and others [see Snyder et al. [25] and Austin et al. [26]] indicate a correlation between posture and sensory processing domains on the HEAT, suggesting that the HEAT could be sensitive enough to identify challenges with sensory processing despite only 20 of the 100 points measuring this domain.

Referring to the occupational therapy case descriptive notes, it was seen that, toward the end of the intervention period, Partic.c's father and younger sibling began attending and watching the treatment session. These occupational therapy case notes were reviewed to provide a therapeutic context for the statistical changes in posture that were measured and for which there was a negative prediction in SPRE. During these last 3-4 sessions, her posture decreased when she looked toward her dad and little sister. Her scores on static posture declined (though the distribution of all HEAT 14 scores was normal with outliers). Her scores on HEAT sensory processing also decreased at session five of the 14 sessions.

Analyzing the outcomes for this participant using the SPRE results pointed to a relationship between static posture and sensory processing that was then confirmed by research literature. Furthermore, the negative predictive direction from the predictions in the SPRE analysis was explained by the occupational therapy descriptive notes during therapy. In this study, translational research “translates” comparative findings in statistical and data science research results into meaningful occupational therapy research, practice, and a better understanding of outcomes. In this sense, translational research may provide insights into implementing “data to practice” outcomes to produce new treatment options for patients.

## Figures and Tables

**Table 1:** Paired *t*-test results of the HEAT pretest/posttest results.

Items for *T*-tests	*N*	*t*	df	Sig.
HEAT total session 1 versus HEAT total session 14	12	−4.033	11	.002^*∗∗*^
HEAT static posture session 1 versus HEAT static posture session 14	12	−2.000	11	.071
HEAT sensory processing session 1 versus HEAT sensory processing session 14	12	−2.568	11	.026^*∗∗*^
HEAT psychosocial/behavioral session 1 versus HEAT psychosocial/behavioral session 14	12	−4.696	11	.001^*∗∗*^

*∗∗* is used by SPSS to signify statistical significance.

**Table 2:** Analysis of the HEATTOTAL of individual participants.

Participant	Domain	Change point number	*R* ^2^	*p* value	Prediction direction	Residuals
Partic.a	Total	14	0.5875	0.00139	Positive	Normal distribution
Partic.b	Total	14	0.8275	0.00000644	Positive	Normal distribution
Partic.c	Total	1	0.2933	0.1656	Positive	Normal distribution
Partic.d	Total	13	0.7777	0.00000303	Positive	Normal distribution
Partic.e	Total	1	0.4153	0.4298	Negative	Normal distribution

**Table 3:** SPRE analysis of 5 participants for the static posture variable.

Participant	Domain	Change point number	*R* ^2^	*p* value	Prediction direction	Residuals
Partic.a	Static	14	0.524	*0.0034*	Positive	Normal distribution
Partic.b	Static	14	0.235	0.0788	Positive	Normal distribution
Partic.c	Static	13	0.454	0.212	Negative	~normal/outliers
Partic.d	Static	14	0.9996	*0.0125*	Positive	Normal distribution
Partic.e	Static	13 (of 13)	0.1282	0.2296	Negative	Normal distribution

**Table 4:** SPRE analysis of one participant for each variable.

Participant	Domain	Change point number	*R* ^2^	*p* value	Prediction direction	Residuals
Partic.c	Static	13	0.454	0.212	Negative	~normal/ladder
Partic.c	Dynamic	13	0.222	0.238	Positive	Normal
Partic.c	Sensory processing	5	0.328	0.065	Positive	Nonnormal/outliers
Partic.c	Psych/Soc	3	0.548	0.153	Positive	Normal

## References

[1] Dunton W. R. (1934). The need for and the value of research in occupational therapy. *Occupational Therapy and Rehabilitation*.

[2] Weissman-Miller D., Shotwell M. P., Miller R. J. (2012). New single-subject and small-n design in occupational therapy: application to weight loss in obesity. *American Journal of Occupational Therapy*.

[3] Weissman-Miller D. (2013). Novel point estimation from a semiparametric ratio estimator (SPRE): long-term health outcomes from short-term linear data, with application to weight loss in obesity. *The International Journal of Biostatistics*.

[4] Weissman-Miller D. (2016). On predicting survival in prostate cancer: using an extended maximum spacing method at the change point of the semiparametric ratio estimator (SPRE). *International Journal of Statistics and Probability*.

[5] American Hippotherapy Association (AHA) http://www.americanhippotherapyassociation.org/.

[6] Granados A. C., Agís I. F. (2011). Why children with special needs feel better with hippotherapy sessions: a conceptual review. *Journal of Alternative and Complementary Medicine*.

[7] Debuse D., Chandler C., Gibb C. (2005). An exploration of German and British physiotherapists' views on the effects of hippotherapy and their measurement. *Physiotherapy Theory and Practice*.

[8] Silkwood-Sherer D. J., Killian C. B., Long T. M., Martin K. S. (2012). Hippotherapy—an intervention to habilitate balance deficits in children with movement disorders: a clinical trial. *Physical Therapy*.

[9] Hamill D., Washington K. A., White O. R. (2007). The effect of hippotherapy on postural control in sitting for children with cerebral palsy. *Physical & Occupational Therapy in Pediatrics*.

[10] Bertoti D. B. (1988). Effect of therapeutic horseback riding on posture in children with cerebral palsy. *Physical Therapy*.

[11] Holm M. B., Baird J. M., Kim Y. J. (2014). Therapeutic horseback riding outcomes of parent-identified goals for children with autism spectrum disorder: an ABA′ multiple case design examining dosing and generalization to the home and community. *Journal of Autism and Developmental Disorders*.

[12] Bass M. M., Duchowny C. A., Llabre M. M. (2009). The effect of therapeutic horseback riding on social functioning in children with autism. *Journal of Autism and Developmental Disorders*.

[13] Park E. S., Rha D.-W., Shin J. S., Kim S., Jung S. (2014). Effects of hippotherapy on gross motor function and functional performance of children with cerebral palsy. *Yonsei Medical Journal*.

[14] Malone T., Maney E., MacSpadden C., Moss E., O'Kelley B., Post E. (2016). *Predictive validity of the Hippotherapy Evaluation and Assessment Tool (HEAT) [M.S. thesis]*.

[15] R Development Core Team (2010). *R: A Language and Environment for Statistical Computing*.

[16] Microsoft Excel (2016). *Microsoft Corporation*.

[17] Weisberg S. (2005). *Applied Linear Regression*.

[18] Weissman-Miller D., Holmes W. (2015). Novel low-error interpolation method for a fall prevention program using the single- subject design statistical model SPRE. *International Journal of Current Research in Life Sciences*.

[19] Cox H., Morgan T., Smith E., Wiles R. (2011). *Effects of hippotherapy on people with cerebral palsy from the users' perspective: a qualitative study [M.S. thesis]*.

[20] Portney L. G., Watkins M. P., Portney L. G., Watkins M. P. (2009). Validity of measurements. *Foundations of Clinical Research: Applications to Practice*.

[21] IBM (2013). *IBM SPSS Statistics for Windows, Version 22.0*.

[22] R Studio v.0.96 http://www.rstudio.com.

[23] Parnell C. St. Andrews University, Numerical Analysis. http://www-solar.mcs.st-andrews.ac.uk/~clare/Lectures/num-analysis/Numan_chap3.pdf.

[24] Case-Smith J., Case-Smith J., O'Brien J. C. (2010). Development of childhood occupations. *Occupational Therapy for Children*.

[25] Snyder J., Smith D., Mapp K., Wade K. (2012). *Establishing concurrent validity of the hippotherapy assessment and evaluation tool [M.S. thesis]*.

[26] Austin A., Bridges K., Pledger D., Truitt L. (2013). *Concurrent validity of the hippotherapy assessment and evaluation tool [M.S. thesis]*.

